# *CTLA4* Single-Nucleotide Polymorphisms Influence the Risk of HSV and VZV Infection in Kidney Transplant Recipients: A Prospective Cohort Study

**DOI:** 10.3389/ti.2025.14648

**Published:** 2025-05-21

**Authors:** Natalia Redondo, Isabel Rodríguez-Goncer, Tamara Ruiz-Merlo, Francisco López-Medrano, Esther González, Natalia Polanco, Ana Hernández-Vicente, Rafael San Juan, Amado Andrés, José María Aguado, Mario Fernández-Ruiz

**Affiliations:** ^1^ Unit of Infectious Diseases, Hospital Universitario “12 de Octubre”, Instituto de Investigación Sanitaria Hospital “12 de Octubre” (imas12), Madrid, Spain; ^2^ Centro de Investigación Biomédica en Red de Enfermedades Infecciosas (CIBERINFEC), Madrid, Spain; ^3^ Department of Medicine, School of Medicine, Universidad Complutense, Madrid, Spain; ^4^ Department of Nephrology, Hospital Universitario “12 de Octubre”, Instituto de Investigación Sanitaria Hospital “12 de Octubre” (imas12), Madrid, Spain

**Keywords:** herpesvirus, kidney transplantation, single-nucleotide polymorphism, cytotoxic T-lymphocyte antigen 4, HSV

## Abstract

Herpesviruses are able to modulate adaptive T-cell-mediated responses to establish latency within the host. Reactivation of herpes simplex virus (HSV)-1/2 and varicella zoster virus (VZV) is a frequent and potentially serious complication among kidney transplant recipients (KTRs). The ability of clinical criteria to identify KTRs at increased risk of α-herpesvirus (HSV/VZV) infection is limited. We investigated the effect of two single nucleotide polymorphisms (SNPs) in the cytotoxic T-lymphocyte antigen 4 *(CTLA4)* gene in a single-center cohort of 204 KTRs. After a median follow-up of 3.1 years, 34 of them (16.7%) experienced 22 episodes of zoster and 15 episodes of HSV-1/2 infection. Homozygous carriers of the minor allele of rs231775 had a higher cumulative incidence of α-herpesvirus infection (23.5% for GG versus 7.6% for AA/AG carriers; *P*-value = 0.011) and a lower infection-free survival (log-rank *P*-value = 0.037). After multivariable adjustment by clinical factors (including use of valganciclovir prophylaxis and acute rejection as time-dependent variables), the GG genotype of *CTLA4* (rs231775) SNP was associated to the study outcome (adjusted hazard ratio: 3.21; 95% confidence interval: 1.44–7.16). In conclusion, genetic polymorphisms in the co-inhibitory T-cell receptor CTLA-4 may be detrimental for the immune control of latent HSV/VZV infection in KTRs.

## Graphical Abstract

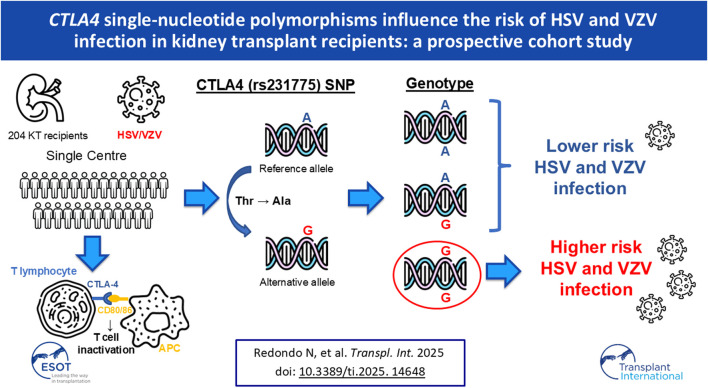

## Introduction

Herpes simplex viruses type 1 and 2 (HSV-1/2) and varicella zoster virus (VZV) are ubiquitous α-herpesviruses able to establish life-long infection and to reactivate under certain circumstances, such as immunosuppression. Solid organ transplant (SOT) recipients are more prone to experiencing reactivation of α-herpesviruses as compared to the general population. The clinical spectrum may range from minor mucocutaneous forms—orolabial or genital vesicular lesions or localized herpes zoster (HZ)— to disseminated disease with central nervous system (CNS) and visceral involvement [1–3]. In addition to older age, use of valganciclovir as prophylaxis against cytomegalovirus (CMV) and increase of immunosuppressive therapy due to previous rejection episodes [1, 4–6], the factors governing the development of post-transplant HSV/VZV reactivation remain poorly characterized.

Single nucleotide polymorphisms (SNP) in genes coding for immune molecules confer a differential susceptibility to viral pathogens. The contribution of host genetics is highlighted after SOT due to the additive effect of iatrogenic immunosuppression. Therefore, SNP genotyping has emerged as a complementary tool for risk stratification in this population [7].

Herpesviruses are able to modulate adaptive T-cell-mediated responses to maintain latency, with CMV as the most notorious example [8]. The expression of co-inhibitory T-cell receptors plays a relevant role in the virus-host interaction [9–11]. Cytotoxic T-lymphocyte antigen 4 (CTLA-4) and its homologous CD28 are two immunoglobulin superfamily members with a shared ability to bind CD80/B7.1 and CD86/B7.2 but opposed biological functions. CTLA-4 suppresses T-cell receptor signaling, contracts the expanded T-cell populations by inhibiting T-cell proliferation and interleukin-2 secretion, and promotes the suppressive functions of Tregs [12–14]. Different SNPs in the *CTLA4* gene have been accordingly investigated in the context of cancer or autoimmune diseases [15] or infection, such as hepatitis C [16] or dengue [17].

The effect of genetic polymorphisms in *CTLA4* on the risk of infectious complications in the specific setting of SOT has been assessed in some previous studies [18–21]. Jiang et al. reported a protective role for the GG genotype of rs231775 on the recurrence of hepatitis B virus infection after liver transplantation [19]. Other study revealed that the presence of the mutant genotypes of rs231775 and rs3087243 were associated with a lower CMV disease-free survival in kidney transplant recipients (KTRs) as compared with heterozygous and wild genotypes [20]. In addition, a meta-analysis established a correlation between two *CTLA-4* SNPs and the risk of post-transplant infection [21]. Of note, this previous research yielded some discrepant results in the sense of the association found (protective or deleterious).

Due to the alleged impact of *CTLA4* SNPs on the host susceptibility and the lack of specific data, we aimed to explore the effect of two *CTLA-4* SNPs (rs5742909 and rs231775) on the incidence of α-herpesvirus infection (HSV/VZV) in a cohort of KTRs.

## Materials and Methods

### Study Population and Design

The present study was based on a prospectively maintained cohort of consecutive KTRs at our institution between November 2014 and December 2016 [22]. The research was performed in accordance with the ethical standards outlined in the Declarations of Helsinki and Istanbul. All the patients provided informed consent and the local Clinical Research Ethics Committee approved the study protocol (number 14/030). The project was developed according to the STREGA statement recommendations.

The study outcome was the occurrence of α-herpesvirus (HSV-1/2 and VZV) infection during the follow-up period. Participants were enrolled at the time of transplantation and followed-up until graft loss, death or December 2018, whichever occurred earlier. None of the included KTRs received the HZ subunit vaccine (HZ/su) during the study period, since this product was approved in Spain in 2020. Descriptions of immunosuppression and prophylaxis regimens are provided as Supplementary Methods. Attending physicians were not made aware of the genotyping results.

### Study Definitions

Mucocutaneous HSV-1/2 infection was diagnosed by the presence of painful vesicular or ulcerative lesions on orolabial, genital or perianal areas, with or without confirmation by polymerase chain reaction (PCR), cell culture or immunohistochemistry (IHC). The diagnosis of visceral disease required compatible clinical manifestations involving the gastrointestinal tract (esophagitis, gastritis or hepatitis), ocular structures (conjunctivitis, keratitis or uveitis) or CNS (meningitis, encephalitis or stroke) associated to a positive result of PCR assay, culture or IHC in an appropriate sample [1]. The diagnosis of HZ was also clinical (characteristic pruritic papulovesicular rash with a dermatomal distribution), and virological or IHC confirmation was not required. Disseminated HZ was defined by lesions involving ≥2 non-contiguous dermatomes or varicella-like syndrome. Complicated HZ comprised ocular or CNS disease or any other visceral involvement with virological and/or IHC documentation [3]. Clinical diagnoses were made by transplant nephrologists, ID physicians or general practitioners (GPs) (with subsequent reevaluation at the transplant outpatient clinic). Additional definitions are available in Supplementary Methods.

### CTLA4 SNP Genotyping

Genotyping was retrospectively performed from whole blood specimens collected at inclusion and stored at −80°C until analysis. DNA was extracted with the KingFisher™ Duo Prime system (Thermo Fisher Scientific, Waltham, MA) using the MagMax™ DNA Multi-Sample Ultra 2.0 kit, following the manufacturer´s instructions. *CTLA4* (rs5742909, rs231775) genotyping was performed by TaqMan technology (Thermo Fisher Scientific) in a QuantStudio 3 system (Applied Biosystems, Foster City, CA). SNP and allele (genotype) calling was made by a standard end-point analysis with the aid of a commercial genotype-calling software (TaqMan™ Genotyper Software v1.0.1) and the QuantStudio Design and Analysis Software v1.5.1 (both from Applied Biosystems).

### Statistical Analysis

Quantitative data were shown as the mean ± standard deviation (SD) or the median with interquartile range (IQR). Deviation from the Hardy-Weinberg equilibrium for each SNP was evaluated by the χ^2^ test with one degree of freedom. Comparisons of the cumulative incidence of α-herpesvirus infection according to the different SNP alleles or genotypes, either individually or in combination, were performed by the χ^2^ test or the Fisher’s exact test. Incidence rates per 1,000 patient-days and the corresponding incidence rate ratio (IRR) were calculated with 95% confidence interval (95 CIs). Survival probabilities were estimated by the Kaplan-Meier method and differences between groups were compared by the log-rank test. Univariable Cox regression was used to identify variables with *P*-value < 0.09, which were entered into a multivariable model that included the selected *CTLA4* SNP as the variable of interest. The exposure to valganciclovir prophylaxis and the occurrence of acute rejection were entered as time-dependent covariates. Since the completeness of the institutional database was very high, no imputation for missing data was performed. Statistical analysis was performed using SPSS v21 (Statistical Package for Social Sciences, Chicago, IL).

## Results

### Study Cohort and Outcomes

We included 204 KTRs (Table 1). After a median follow-up period of 3.1 years (IQR: 2.6–3.6), 34 patients (16.7%) developed 37 episodes of α-herpesvirus infection, yielding an incidence rate of 0.17 cases per 1,000 patient-days (95% CI: 0.12–0.23). The median interval between transplantation and the first episode was 454.5 days (IQR: 47.5–1639.8). In detail, 16.2% (6/37), 21.6% (8/37) and 62.2% (23/37) of episodes occurred in the early (first month), intermediate (1–6 moths) and late post-transplant periods (≥6 months), respectively.

**TABLE 1:** Demographics and clinical characteristics of the study cohort (n = 204).

Variable	
Age, years [mean ± SD]	54.6 ± 15.7
Gender (male) [n (%)]	146 (71.6)
Body mass index, kg/m^2^ [mean ± SD]	25.9 ± 9.5
Ethnicity [n (%)]	
Caucasian	177 (86.8)
Hispanic	17 (8.3)
African	6 (2.9)
Asian	4 (2.0)
Current or prior smoking history [n (%)]	81 (39.9)
Pre-transplant chronic co-morbidities [n (%)]	
Hypertension	175 (85.8)
Diabetes mellitus	58 (28.4)
Non-coronary heart disease	35 (17.2)
Chronic lung disease	27 (13.2)
Coronary heart disease	21 (10.3)
Peripheral arterial disease	21 (10.3)
Solid or hematological malignancy or melanoma	20 (9.8)
Previous solid organ transplantation [n (%)]	28 (13.7)
Underlying end-stage renal disease [n (%)]	
Diabetic nephropathy	35 (17.2)
Glomerulonephritis	55 (27.0)
Polycystic kidney disease	24 (11.8)
Hypertensive nephropathy	18 (8.8)
Congenital nephropathy	8 (3.9)
Reflux nephropathy	7 (3.4)
Unknown	25 (12.3)
Other	32 (15.7)
CMV serostatus [n (%)]	
D+/R+	148 (72.5)
D+/R-	23 (11.3)
D-/R+	22 (10.8)
D-/R-	7 (3.4)
D unknown/R+	4 (2.0)
Positive HCV serostatus [n (%)]^a^	15 (7.4)
Positive HIV serostatus [n (%)]^b^	2 (1.0)
Positive VZV serostatus [n (%)]^c^	186 (95.4)
Pre-transplant renal replacement therapy [n (%)]	180 (88.2)
Hemodialysis	148/180 (82.2)
Continuous ambulatory peritoneal dialysis	32/180 (17.8)
Time on dialysis, months [median (IQR)]	17.2 (8.9–35.4)
Age of donor, years [mean ± SD]	53.8 ± 15.5
Gender of donor (male) [n (%)]	109 (53.4)
Type of donor [n (%)]	
DBD donor	128 (62.7)
DCD donor	46 (22.6)
Living donor	29 (14.2)
Cold ischemia time, hours [median (IQR)]	18.0 (10.1–23.0)
Number of HLA mismatches [median (IQR)]	4 (3–5)
Induction therapy [n (%)]	
ATG	94 (46.1)
Basiliximab	83 (46.7)
None	27 (13.2)
Primary immunosuppression regimen [n (%)]	
Prednisone, tacrolimus and MMF/MPS	196 (96.1)
Prednisone, tacrolimus and azathioprine	8 (3.9)
Conversion to mTOR inhibitor during follow-up [n (%)]	19 (9.3)
Time to conversion, days [median (IQR)]	232 (118–321)
Anti-CMV prophylaxis with valganciclovir [n (%)]	113 (55.4)
Duration of prophylaxis, days [median (IQR)]	103 (91–147)
Post-transplant complications [n (%)]	
Delayed graft function	99 (48.5)
New-onset diabetes	24 (11.8)
CMV infection [n (%)]	114 (55.9)
CMV disease [n (%)]	22 (10.8)
Renal artery stenosis	40 (19.6)
Acute graft rejection	25 (12.3)
Time to the first episode, days [median (IQR]	134 (28.5–291.5)
T-cell-mediated acute rejection	16 (7.8)
Borderline T-cell-mediated rejection	8 (3.9)
Antibody-mediated acute rejection	5 (2.5)
Graft loss	8 (3.9)
All-cause death	11 (5.4)

ATG, antithymocyte globulin; CMV, cytomegalovirus; D, donor; DBD, donation after brain death; DCD, donation after circulatory death; HCV, hepatitis C virus; HIV, human immunodeficiency virus; HLA, human leukocyte antigen; IQR, interquartile range; MMF/MPS, mycophenolate mofetil/mycophenolate sodium; mTOR, mammalian target of rapamycin; R, recipient; SD, standard deviation; VZV, varicella zoster virus.

^a^ At the pre-transplant evaluation. Data not available for 5 patients.

^b^ At the pre-transplant evaluation. Data not available for 2 patients.

^c^ At the pre-transplant evaluation. Data not available for 7 patients.

There were 22 episodes of VZV infection in form of HZ confined to a single dermatome, the diagnosis of which was based solely on clinical manifestations. All of them occurred in KTRs that were VZV-seropositive before transplantation. The 15 episodes of HSV-1/2 infection included mucocutaneous disease in form of orolabial (9 cases) or genital herpes (4 cases), HSV esophagitis and HSV pharyngitis with facial palsy (one case each). There were no cases of visceral or disseminated disease. The diagnosis of HSV-1/2 infection was confirmed by cell culture (5/15 [33.3%]), IHC (2/15 [13.3%]) or clinical findings alone (8/15 [53.3%]).

### Association Between *CTLA4* SNPs and α-Herpesvirus Infection

All the SNP genotype frequencies were in Hardy-Weinberg equilibrium (data not shown). First, we investigated whether the presence of specific alleles within the *CTLA4* gene was correlated with the cumulative incidence of α-herpesvirus infection. There were no significant differences in the allele distribution of the *CTLA4* (rs5742909) SNP between KTRs that experienced or did not experience the study outcome (*P*-value = 0.967). In contrast, the presence of the minor allele G of the *CTLA4* (rs231775) SNP was significantly more common among KTRs with α-herpesvirus infection *(P*-value = 0.005) (Supplementary Table S1 of Supplementary Material). Subsequently, we tested both dominant and recessive models. Only carriers of the G allele in a homozygous state experienced a higher incidence of infection (23.5% [8/34] for GG versus 7.6% [13/170] for AA/AG; *P*-value = 0.011), suggesting a recessive effect (Table 2). The incidence rates were 0.375 (95% CI: 0.180–0.690) and 0.137 (95% CI: 0.180–0.690) episodes per 1,000 patient-days for the GG and AA/AG genotypes, respectively *(P-*value = 0.004), with an IRR of 2.73 (95% CI: 1.18–5.82; *P*-value = 0.013). Time-to-event Kaplan-Meier curves for time to first episode of α-herpesvirus infection according to the genotype of rs231775 are shown in Supplementary Figure S1. There were no differences in the length of follow-up according to the genotype (median of 3.5 [IQR: 1.7–3.8] years for the GG genotype versus 3.0 [IQR: 2.6–3.6] years for AA/AG genotypes; *P*-value = 0.848).

**TABLE 2:** Cumulative incidence of α-herpesvirus infection according to dominant and recessive models for candidate *CTLA4* SNPs.

Gene (SNP database ID number)	Model	Genotype	α-herpesvirus infection, n (%)		*P*-value
			No (n = 170)	Yes (n = 34)	
*CTLA4* (rs5742909)	Dominant	CC	139 (81.8)	28 (82.4)	0.935
		CT/TT	31 (18.2)	6 (17.6)	
	Recessive	CC/CT	166 (97.6)	33 (97.1)	1.000
		TT	4 (2.6)	1 (2.9)	
*CTLA4* (rs231775)	Dominant	AA	85 (50.0)	19 (55.9)	0.531
		AG/GG	85 (50.0)	15 (44.1)	
	Recessive	AA/AG	157 (92.4)	26 (76.5)	0.011
		GG	13 (7.6)	8 (23.5)	

ID, identification; SNP, single-nucleotide polymorphism; CTLA, cytotoxic T-lymphocyte antigen.

### α-Herpesvirus Infection-Free Survival

**FIGURE 1:**
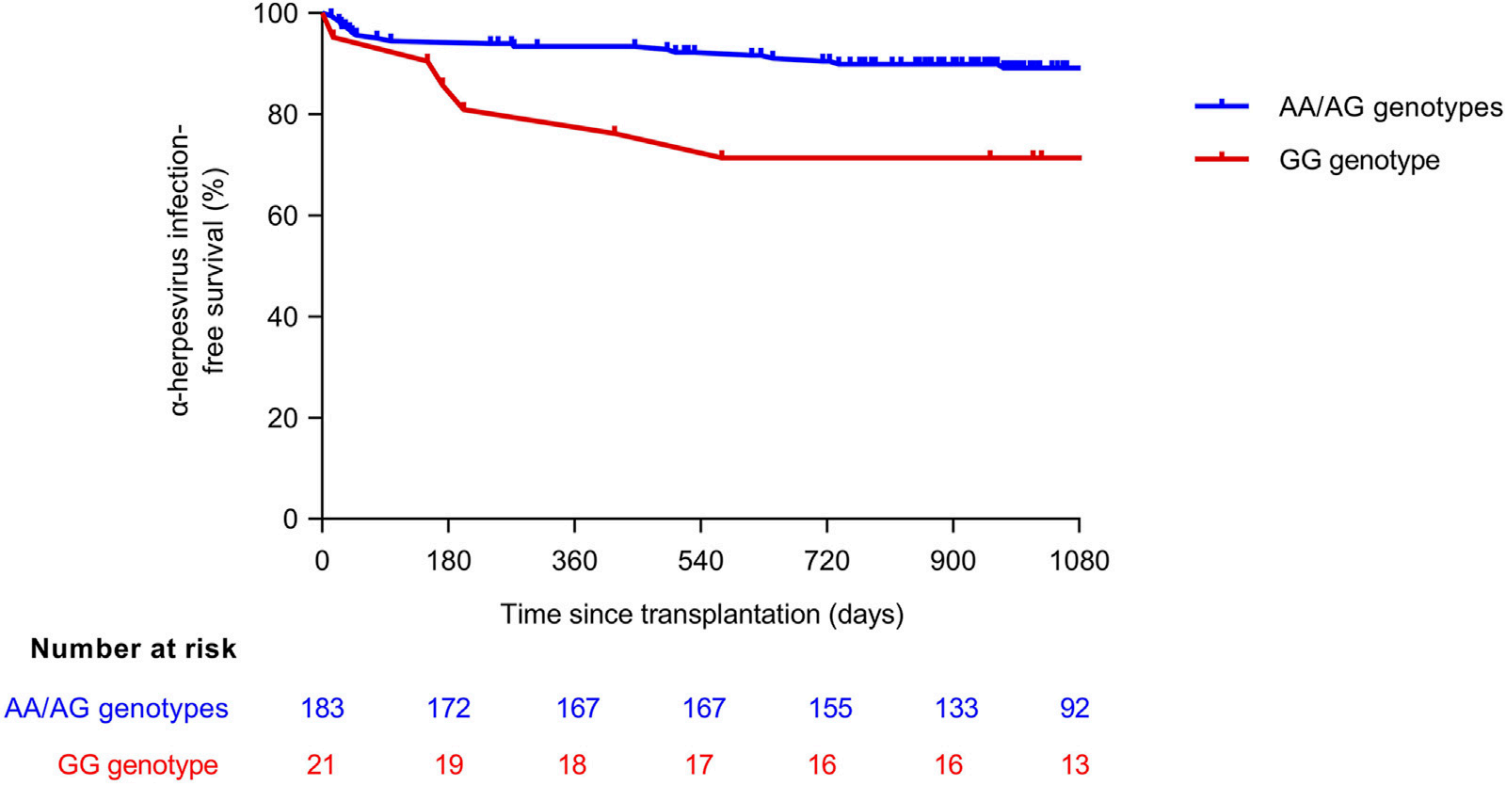
Kaplan-Meier α-herpesvirus infection-free survival curves according to the genotype of *CTLA4* (rs231775) SNP (log-rank *P*-value = 0.037). CTLA, cytotoxic T-lymphocyte antigen; SNP, single-nucleotide polymorphism.

We plotted α-herpesvirus infection-survival curves according to the genotype of rs231775 (Figure 1). KTRs that were homozygous or heterozygous for the reference allele (AA/AG) were significantly more likely to remain free from infection as compared to GG carriers (log-rank *P*-value = 0.037). After multivariable adjustment by gender, pre-transplant diabetes mellitus, use of valganciclovir as CMV prophylaxis, cold ischemia time and occurrence of acute rejection, the GG genotype of *CTLA4* (rs231775) SNP remained associated to α-herpesvirus infection (adjusted hazard ratio: 3.21; 95% CI: 1.44–7.16; *P*-value = 0.004) (Table 3).

**TABLE 3:** Univariable and multivariable Cox regression models to predict the occurrence of α-herpesvirus infection.

	No α-herpesvirus infection (n = 170)	α-herpesvirus infection (n = 34)	*P*-value	Univariable analysis			Multivariable analysis		
				HR	95% CI	*P-*value	HR	95% CI	*P-*value
Age of recipient, years [mean ± SD]	54.7 ± 15.9	54.1 ± 14.9	0.411	1.05^a^	0.83–1.33	0.675			
Gender (male) [n (%)]	117 (68.8)	29 (85.3)	0.052	4.86	1.15–20.59	0.032	2.24	0.84–5.98	0.107
Body mass index, kg/m^2^ [mean ± SD]	25.2 ± 3.7	26.0 ± 10.4	0.623	0.99^b^	0.95–1.04	0.883			
Non-Caucasian ethnicity [n (%)]	150 (88.2)	27 (79.4)	0.172	0.55	0.21–1.46	0.231			
Current or prior smoking history [n (%)]	68 (40.0)	13 (38.2)	0.848	1.04	0.47–2.31	0.929			
Pre-transplant hypertension [n (%)]	146 (86.4)	29 (85.3)	0.791	1.15	0.33–3.84	0.821			
Pre-transplant diabetes mellitus [n (%)]	47 (27.6)	11 (32.4)	0.579	2.18	0.99–4.79	0.054	1.05	0.49–2.26	0.905
Pre-transplant coronary heart disease [n (%)]	16 (9.4)	5 (14.7)	0.358	1.95	0.74–5.13	0.178			
Pre-transplant chronic lung disease [n (%)]	20 (11.8)	7 (20.6)	0.172	2.20	0.88–5.51	0.092			
Pre-transplant peripheral arterial disease [n (%)]	18 (10.6)	3 (8.8)	1.000	1.33	0.39–4.45	0.641			
Pre-transplant malignancy [n (%)]	16 (9.4)	4 (11.8)	0.751	1.79	0.62–5.23	0.284			
Pre-transplant renal replacement therapy [n (%)]	149 (87.6)	31 (91.2)	0.772	0.98	0.29–3.27	0.972			
Time on dialysis, months [median (IQR)]	17.3 (8.8–35.3)	13.1 (9.3–35.9)	0.950	0.99^b^	0.99–1.01	0.384			
Diabetic nephropathy as ESRD [n (%)]	31 (18.2)	4 (11.8)	0.361	0.97	0.33–2.82	0.954			
Glomerulonephritis as ESRD [n (%)]	44 (25.9)	11 (32.4)	0.438	1.06	0.44–2.55	0.890			
Polycystic kidney disease as ESRD [n (%)]	22 (12.9)	2 (5.9)	0.382	0.30	0.04–2.22	0.238			
Previous solid organ transplantation [n (%)]	22 (12.9)	6 (17.6)	0.426	1.07	0.43–2.63	0.887			
Mismatched CMV serostatus (D+/R-) [n (%)]	19 (11.3)	4 (12.5)	0.769	1.11	0.39–3.21	0.846			
Positive CMV serostatus (R+) [n (%)]	145 (85.3)	30 (88.2)	0.792	1.97	0.47–8.37	0.356			
Positive HCV serostatus (R+) [n (%)]	11 (6.6)	4 (12.1)	0.281	1.83	0.55–6.14	0.327			
Positive VZV serostatus (R+) [n (%)]	154 (94.5)	32 (100.0)	0.360	21.73	0.01–723, 315	0.457			
Age of donor, years [mean ± SD]	53.7 ± 15.4	54.4 ± 16.4	0.400	1.98^a^	0.87–1.38	0.432			
DCD donor [n (%)]	40 (23.5)	6 (17.6)	0.454	0.88	0.36–2.16	0.779			
Living donor [n (%)]	26 (15.3)	3 (8.8)	0.426	0.24	0.03–1.74	0.156			
Cold ischemia time, hours [median (IQR)]	17.3 (9.1–22.3)	19 (13.7–23.1)	0.148	1.07^b^	1.02–1.14	0.013	1.04	0.99–1.09	0.083
Number of HLA mismatches [median (IQR)]	4 (3–5)	5 (3–5.3)	0.340	1.17^b^	0.86–1.58	0.326			
Induction therapy with ATG [n (%)]	77 (45.3)	17 (50.0)	0.615	0.81	0.40–1.65	0.569			
Induction therapy with basiliximab [n (%)]	69 (40.6)	14 (41.2)	0.949	1.17	0.53–2.58	0.694			
No induction therapy [n (%)]	24 (14.1)	3 (8.8)	0.581	0.61	0.19–1.99	0.410			
CMV antiviral prophylaxis [n (%)]^c^	94 (55.3)	19 (55.9)	0.950	0.32	0.09–1.18	0.088	0.29	0.08–1.11	0.071
PBLSs at month 1, x 10^3^ cells/μL [median (IQR)]									
CD3^+^ T-cell count	0.857 (0.306–1.443)	0.673 (0.194–1.317)	0.363	1.00^b^	0.99–1.00	0.517			
CD4^+^ T-cell count	0.495 (0.155–0.991)	0.378 (0.127–0.754)	0.273	1.00^b^	0.99–1.00	0.302			
CD8^+^ T-cell count	0.278 (0.129–0.278)	0.273 (0.101–0.538)	0.763	1.00^b^	0.99–1.00	0.745			
Acute rejection during the first 12 months [n (%)]^c^	18 (10.6)	4 (11.8)	0.768	7.12	1.58–32.58	0.011	7.79	1.67–36.31	0.009
GG genotype of *CTLA4* (rs231775) SNP [n (%)]	13 (7.6)	8 (23.5)	0.011	2.95	1.18–7.39	0.021	3.21	1.44–7.16	0.004

ATG, antithymocyte globulin; CMV, cytomegalovirus; D, donor; DCD, donation after circulatory death; ESRD, end-stage renal disease; HCV, hepatitis C virus; HR, hazard ratio; IQR, interquartile range; PBLSs, peripheral blood lymphocyte subpopulations; R, recipient; SD, standard deviation; VZV, varicella zoster virus.

^a^ HR per 10-year increment.

^b^ HR per unitary increment.

^c^ Time-dependent covariate.

## Discussion

We have shown an association between the presence of the minor allele of rs231775 in the *CTLA4* gene and the susceptibility to α-herpesviruses in KTRs. Homozygous carriers of the G allele faced a more than three-fold increase in the incidence of HSV-1/2 and VZV infection, typically in form of mucocutaneous disease and unidermatomal HZ secondary to viral reactivation. This impact was still significant after controlling for well-established risk factors, such as valganciclovir prophylaxis or over-immunosuppression due to recent treatment for acute rejection [1, 4–6].

The *CTLA4* (rs231775) SNP consists of a nonsynonymous A/G substitution that implies the change from threonine to alanine, which lead to lower expression levels of membrane-bound CTLA-4 [23, 24]. In keeping with this effect, the GG genotype has been associated with a lower mortality in sepsis patients, a finding presumably attributable to a less pronounced sepsis-associated immunoparalysis [25]. Limited evidence is available regarding the risk of post-transplant infection. In pediatric heart transplant recipients, Ohman et al. reported a significant (albeit modest) association between AA/AG genotypes and the late occurrence of viral infection at the univariable level, but not in the adjusted Cox model. Although the authors did not provided data on specific viral pathogens, it is likely that most episodes were due to primary infection rather than reactivation, in view of the age of the cohort [26]. Iravani Saadi et al. performed a meta-analysis on the basis of 9 studies that found a protective effect for the A allele of the rs231775 SNP (odds ratio: 0.77; 95% CI: 0.59–0.95). Unfortunately, no details on the type of SOT or infection were provided, or whether the genotyping was performed in the donor or the recipient, which limited the possibility of drawing clear conclusions [21].

We are not aware of previous studies that have investigated the effect of genetic polymorphisms in *CTLA4* on the risk of post-transplant HSV-1/2 or VZV infection. Thus, the present results should be considered hypothesis-generating only. The mechanistic explanation is not straightforward, since the rs231775 G allele has been shown to reduce the inhibitory function of CTLA-4 through decreased cell surface expression and ligand affinity [23, 24]. This should result in the improved immune control of latent α-herpesvirus infection. Nevertheless, the frequency of the GG genotype of *CTLA4* (rs4553808) SNP—which is mapped within the promoter region and also alters its transcription rate—was significantly higher in Chinese KTRs that developed viral infection as compared to those without [18]. It may hypothesized that a lower baseline CTLA-4 expression on polyclonal activated T-cells would render more effective the induction of phenotypically exhausted virus-specific CD8^+^ T-cells, which is one of the immune evasion tactics displayed by HSV and VZV [27, 28]. This susceptibility would be specific for α-herpesviruses, since we have found no association between *CTLA4* SNPs and the incidence of CMV infection (data not shown).

Our study is limited by the relatively low number of KTRs that developed infection and the lack of severe cases. Since no data on the baseline HSV-1/2 serostatus was available, we cannot rule out that some episodes were secondary to primary infection rather than reactivation, which would imply a differential role for virus-induced immune evasion. In addition, we lack granular data on the receipt of immunosuppressive therapy before transplantation. Nevertheless, neither the presence of glomerulonephritis as ESRD nor previous SOT (as two surrogate markers for pre-transplant immunosuppression) had an apparent impact on the event of interest. Most episodes of shingles and orolabial HSV infection were diagnosed solely based on clinical findings, and some of them by GPs (although with prompt referral to the transplant outpatient clinic). However, previous studies have reported that GPs have good clinical judgment for the diagnosis of herpes zoster [29]. Finally, the assessment of the confounding effect associated to the use of valganciclovir prophylaxis may have limited by the relatively low number of KTRs in this subgroup.

Future investigations should provide a functional insight into the immune and cellular mechanisms eventually involved in the association observed between *CTLA4* polymorphisms and susceptibility to α-herpesvirus infection among KTRs. In the current setting of increasing availability of the HZ/su vaccine for the immunocompromised population, it might be worth exploring whether carriers of the risk-genotype would additionally benefit from extended antiviral prophylaxis during the early post-transplant period.

## Data Availability

The raw data supporting the conclusions of this article will be made available by the authors, without undue reservation.

## Abbreviations

CMV, cytomegalovirus; CTLA-4, cytotoxic T-lymphocyte antigen 4; D, donor; GP, general practitioner; HSV, herpes simplex virus; HZ, herpes zoster; HZ/su, HZ subunit vaccine; IQR, interquartile range; IHC, immunohistochemistry; IRR, incidence rate ratio; KTR, kidney transplant recipient; PCR, polymerase chain reaction; R, recipient; PBMC, peripheral mononuclear cell; SD, standard deviation; SNP, single-nucleotide polymorphism; SOT, solid organ transplantation; VZV, varicella-zoster virus.
