# Supplementary material for: CTLA4 Single-Nucleotide Polymorphisms Influence the Risk of HSV and VZV Infection in Kidney Transplant Recipients: A Prospective Cohort Study
Source: Transpl Int. 2025 May 21;38:14648. doi: 10.3389/ti.2025.14648 (PMC12133603; doi:10.3389/ti.2025.14648)
Supplement: Supplementary file 1 [file DataSheet1.docx]

***Supporting Material***

**Supplementary Methods**

*Immunosuppression and prophylaxis regimens*

Induction therapy with intravenous (IV) rabbit antithymocyte globulin (ATG-Fresenius^®^, 1.25 mg/Kg daily for 5-7 days) with delayed initiation of tacrolimus on post-transplant day 6 was used in the case of donation after circulatory death. Patients at high immunological risk also received ATG induction for 1-3 days with immediate tacrolimus initiation. Basiliximab induction (20 mg on days 0 and 4) with delayed tacrolimus introduction on day 5 was reserved to patients at risk for nephrotoxicity (i.e. older age or comorbidities). The standard maintenance immunosuppression regimen consisted of tacrolimus (0.1 mg/Kg daily, adjusted to a target trough level of 10-15 ng/mL during the first month and 5-10 ng/mL thereafter), mycophenolate mofetil (1 g twice daily) or enteric-coated mycophenolate sodium (720 mg twice daily), and prednisone (1 mg/Kg daily with progressive tapering). Conversion to mammalian target of rapamycin inhibitor (typically everolimus) with reduced-dose tacrolimus (target trough level of 3-6 ng/mL) was performed on an individual basis for recipients experiencing tacrolimus-related adverse effects, difficult-to-treat cytomegalovirus (CMV) or BK polyomavirus viremia, or malignancy.

All patients received a single IV dose of cefazolin (or ciprofloxacin in the case of hypersensitivity to ß-lactams) as preoperative antibiotic prophylaxis. Prophylaxis against *Pneumocystis jirovecii* was based on trimethoprim-sulfamethoxazole (160/800 mg three times weekly) or monthly aerosolized pentamidine (300 mg) for 9 months. Patients at high-risk for CMV infection (D+/R- or R+ with ATG induction therapy) received oral valganciclovir (900 mg daily) for 6 or 3 months, respectively. Intermediate-risk patients (R+ without T-cell-depleting therapy) were monitored every 2-4 weeks for CMV viremia with a PCR-based assay and received preemptive therapy with IV ganciclovir (5 mg/Kg twice daily) or oral valganciclovir (900 mg twice daily) for at least 2 weeks in presence of high-level (>1,000 IU/mL) or rapidly increasing viremia (defined by the doubling of DNAemia over two consecutive measurements less than one week apart). Valganciclovir doses were adjusted to renal function according to the manufacturer's recommendations.

*Additional study definitions*

Delayed graft function denoted the requirement for dialysis within the first week. Acute graft rejection was suspected in case of sudden deterioration of graft function and confirmed by graft biopsy examination. Graft loss was defined by the definitive return to dialysis, nephrectomy and/or retransplantation.

**Supplementary Results**

**Table S1.** Cumulative incidence of α-herpesvirus infection according to different genotypes of candidate *CTLA4* SNPs.

| Gene (SNP database ID number) | Genotype | α-herpesvirus infection, n (%) | | *P*-value |
| --- | --- | --- | --- | --- |
|  |  | No (n = 170) | Yes (n = 34) |  |
| *CTLA4* (rs5742909) | CC | 139 (81.8) | 28 (82.4) | 0.967 |
|  | CT | 27 (15.9) | 5 (14.7) |  |
|  | TT | 4 (2.4) | 1 (2.9) |  |
| *CTLA4* (rs231775) | AA | 85 (50.0) | 19 (55.9) | 0.005 |
|  | AG | 72 (42.4) | 7 (20.6) |  |
|  | GG | 13 (7.6) | 8 (23.5) |  |
| ID: identification; SNP: single-nucleotide polymorphism; CTLA: cytotoxic T-lymphocyte antigen. | | | | |


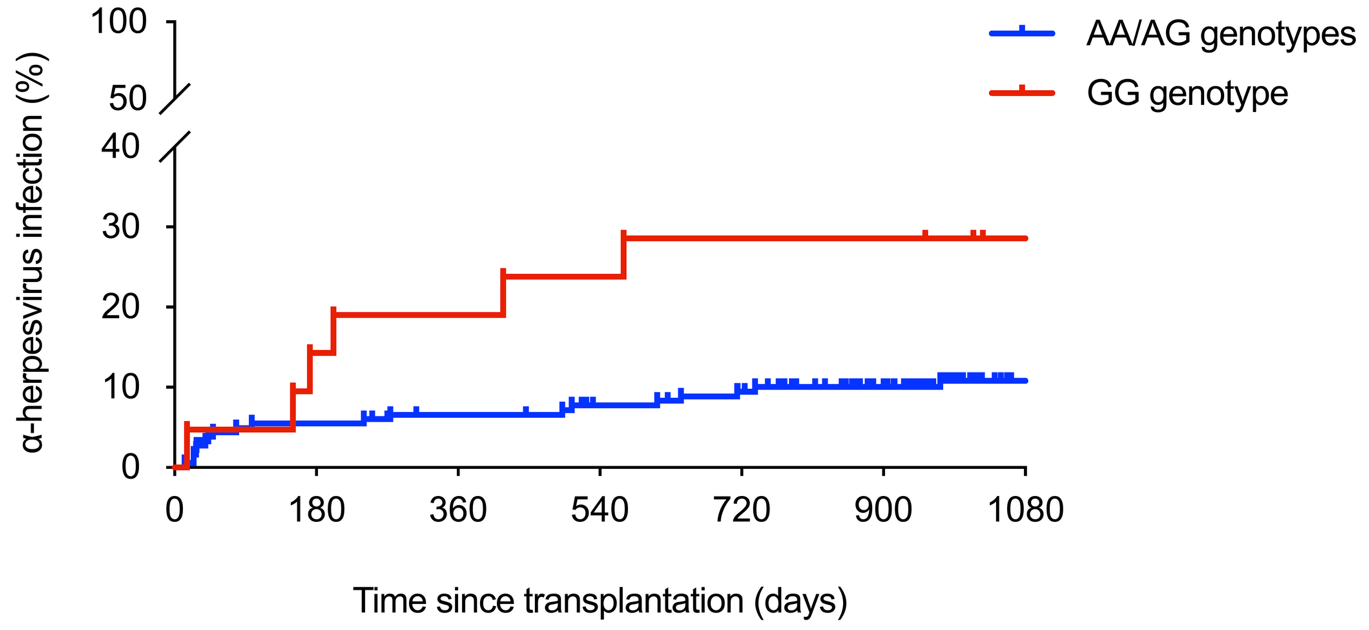
**Figure S1.** Time-to-event Kaplan-Meier curves for time to first episode of α-herpesvirus infection according to the genotype of CTLA4 (rs231775) SNP.
